# Adjuvant chemotherapy compared with observation in patients with T2aN0 stage IB lung adenocarcinoma

**DOI:** 10.3389/fonc.2023.1096683

**Published:** 2023-02-28

**Authors:** Po-Hsin Lee, Chun-Ju Chiang, Jeng-Sen Tseng, Zhe-Rong Zheng, Kun-Chieh Chen, Cheng-Hsiang Chu, Yen-Hsiang Huang, Kuo-Hsuan Hsu, Wen-Chung Lee, Tsung-Ying Yang, Tsang-Wu Liu, Jiun-Yi Hsia, Gee-Chen Chang

**Affiliations:** ^1^ Division of Chest Medicine, Department of Internal Medicine, Taichung Veterans General Hospital, Taichung, Taiwan; ^2^ College of Medicine, National Yang Ming Chiao Tung University, Taipei, Taiwan; ^3^ Ph.D. Program in Translational Medicine, National Chung Hsing University, Taichung, Taiwan; ^4^ Rong Hsing Research Center For Translational Medicine, National Chung Hsing University, Taichung, Taiwan; ^5^ Institute of Epidemiology and Preventive Medicine, College of Public Health, National Taiwan University, Taipei, Taiwan; ^6^ Taiwan Cancer Registry, Taipei, Taiwan; ^7^ Institute of Biomedical Sciences, National Chung Hsing University, Taichung, Taiwan; ^8^ Department of Post-Baccalaureate Medicine, College of Medicine, National Chung Hsing University, Taichung, Taiwan; ^9^ Division of Pulmonary Medicine, Department of Internal Medicine, Chung Shan Medical University Hospital, Taichung, Taiwan; ^10^ School of Medicine, Chung Shan Medical University, Taichung, Taiwan; ^11^ Institute of Medicine, Chung Shan Medical University, Taichung, Taiwan; ^12^ Division of Critical Care and Respiratory Therapy, Department of Internal Medicine, Taichung Veterans General Hospital, Taichung, Taiwan; ^13^ Department of Life Sciences, National Chung Hsing University, Taichung, Taiwan; ^14^ National Institute of Cancer Research, National Health Research Institutes, Miaoli, Taiwan; ^15^ Division of Thoracic Surgery, Department of Surgery, Chung Shan Medical University Hospital, Taichung, Taiwan

**Keywords:** lung adenocarcinoma, adjuvant chemotherapy, T2aN0, stage IB, early lung cancer

## Abstract

**Introduction:**

For patients with T2aN0 stage IB lung adenocarcinoma, benefits of adjuvant chemotherapy remain controversial. Here, we aimed to evaluate such benefits.

**Methods:**

This retrospective cohort study was conducted on the database of the National Taiwan Cancer Registry. We analyzed patients with T2aN0 stage IB lung adenocarcinoma (re-classified by AJCC 8th edition) diagnosed during the period from January 2011 to December 2017. They were divided into two groups: (1) group 1: tumor <=3 cm with visceral pleural invasion (VPI); (2) group 2: tumor >3 cm, but <=4 cm. Overall survival (OS) and cancer specific survival (CSS) were evaluated. Risk factors for survival were determined.

**Results:**

A total of 2,100 patients with T2aN0 stage IB lung adenocarcinoma (1,265 in group 1 and 835 in group 2) were enrolled for study. The proportions of patients receiving adjuvant chemotherapy in group 1 and 2 were 39.1% and 68.6%, respectively. Amongst group 1 patients, adjuvant chemotherapy was not an independent risk factor for OS and CSS. Amongst group 2 patients, high-grade histologic findings and receiving sublobar resection were two risk factors for poorer survival. Adjuvant chemotherapy was also associated with an OS (adjusted hazard ratio (aHR), 0.52; 95% confidence interval (CI), 0.38-0.72; P<0.001) and CSS (aHR, 0.54; 95% CI, 0.37-0.78; p=0.001) benefit regardless of the presence or absence of risk factors.

**Conclusion:**

For patients with T2aN0 stage IB lung adenocarcinoma, adjuvant chemotherapy improved OS and CSS in those with tumors >3 cm, but <=4 cm.For patients with tumors <=3 cm with VPI, adjuvant chemotherapy had no survival benefit.

## Introduction

Lung cancer is by far the leading cause of cancer-related death (1). Complete surgical resection of the tumor provides a hope for a cure for those patients with resectable disease (2). However, post-operative recurrence poses a main problem of the treatment (3). Therefore, identifying populations who may benefit from additional treatment after surgery may improve the clinical outcomes in those patients with resectable lung cancer.

Several randomized clinical trials reported the efficacy of adjuvant chemotherapy following surgery in patients with resectable lung cancer (4–8). The pooled analysis of 5 trials of cisplatin-based adjuvant chemotherapy revealed benefit of adjuvant chemotherapy in completely resected lung cancer patients at an overall hazard ratio (HR) of 0.89 (95% CI, 0.82-0.96; p=0.005). In further subgroup analysis, the benefit is restricted to patients with stage II or IIIA disease. There was no significant improvement of survival in patients with stage IB or IA lung cancer (9). Another study, Cancer and Leukemia Group B (CALGB) 9633, a randomized controlled trial, was designed to solve the unmet need. Patients enrolled had pathologically confirmed T2N0 (according to the International System for Staging Lung Cancer edition in 1997) (10) non-small-cell lung carcinoma (NSCLC) undergoing complete surgical resection. The study showed a significant survival benefit of adjuvant chemotherapy for patients with tumors 4 cm or larger in diameter (HR, 0.69; 95% CI, 0.48- 0.99; p=0.043) (11).

Tumors larger than 4 cm, but 5 cm or less in size without lymph node metastasis are now classified as T2bN0 stage IIA lung cancer, according to AJCC staging system 8^th^ edition (12). Their benefits of adjuvant chemotherapy are mentioned above (11). On the other hand, for patients with T2aN0 stage IB lung cancer, benefits of adjuvant chemotherapy remain unclear. Though several studies advocated the benefit of adjuvant chemotherapy for patients with stage IB lung cancer (8, 11, 13–16). the cancer staging was based on the 5th, 6th, or 7th international staging criteria (10, 17). Furthermore, prior randomized controlled trials enrolled NSCLC patients and did not subdivide them according to histology types. Nevertheless, there is increasing evidence that different histology types (lung adenocarcinoma vs. non-adenocarcinoma) presented with different clinical outcomes (18, 19). A meta-analysis partially answered the above-mentioned questions. The author pooled the studies regarding the impact of adjuvant chemotherapy in stage IB NSCLC in the context of the 8th TNM staging system. Subgroup analysis by histology indicated that adjuvant chemotherapy conferred more favorable survival to both squamous cell carcinoma and adenocarcinoma. However, the eligible studies were retrospective and with population heterogeneity, and subgroup analysis according to tumor size (e.g., tumor <=3 cm vs. tumor >3 but <=4 cm) was not performed (20). Apart from tumor size, other high-risk histopathologic features (e.g., tumor differentiation, vascular invasion, visceral pleural involvement) and surgical factors (e.g., sublobar resection, unknown lymph node status) are presumably indications for adjuvant chemotherapy (21). Little evidence is available to support these indications. Here, we conducted a retrospective cohort study on a nationwide population database in Taiwan, aiming to determine benefits of adjuvant chemotherapy for patients with completely resected T2aN0 stage IB lung adenocarcinoma.

## Materials and methods

### Data source

This retrospective cohort study used data from the National Taiwan Cancer Registry. The database was established by the Ministry of Health and Welfare in 1979, and it kept standardized records of patients’ characteristics and clinical information on all newly diagnosed malignant cancer cases in Taiwan (22–24). Detailed information on the smoking status for lung cancer patients has been recorded in the database starting since 2011. We analyzed newly diagnosed lung cancer patients from January 2011 to December 2017. The main outcome parameter was overall survival and cancer-specific survival. This study was approved by the Research Ethics Committee of the National Taiwan University (NTU-REC No.202101HM030), with waiver of informed consent owing to the lack of personal information and use of secondary data in the study. The Strengthening the Reporting of Observational Studies in Epidemiology (STROBE) reporting guideline for observational studies was used in the revision of this article.

### Data records and definition

Clinical data used for analysis included the following: age at diagnosis, sex, Eastern Cooperative Oncology Group (ECOG) performance status, histologic types, tumor size, tumor stage, smoking status, histologic grade, visceral pleural invasion (VPI), extent of resection, adjuvant treatment, status of N2 stations dissection, and types of health care institution. Sublobar resection refers to wedge resection and segmentectomy. Histologic grade was grouped into low grade (well or moderately differentiated) and high grade (undifferentiated or poorly differentiated). The staging system of lung cancer before 2018 was conducted according to the AJCC staging system 7^th^ edition (17).

### Study population

**Figure 1:**
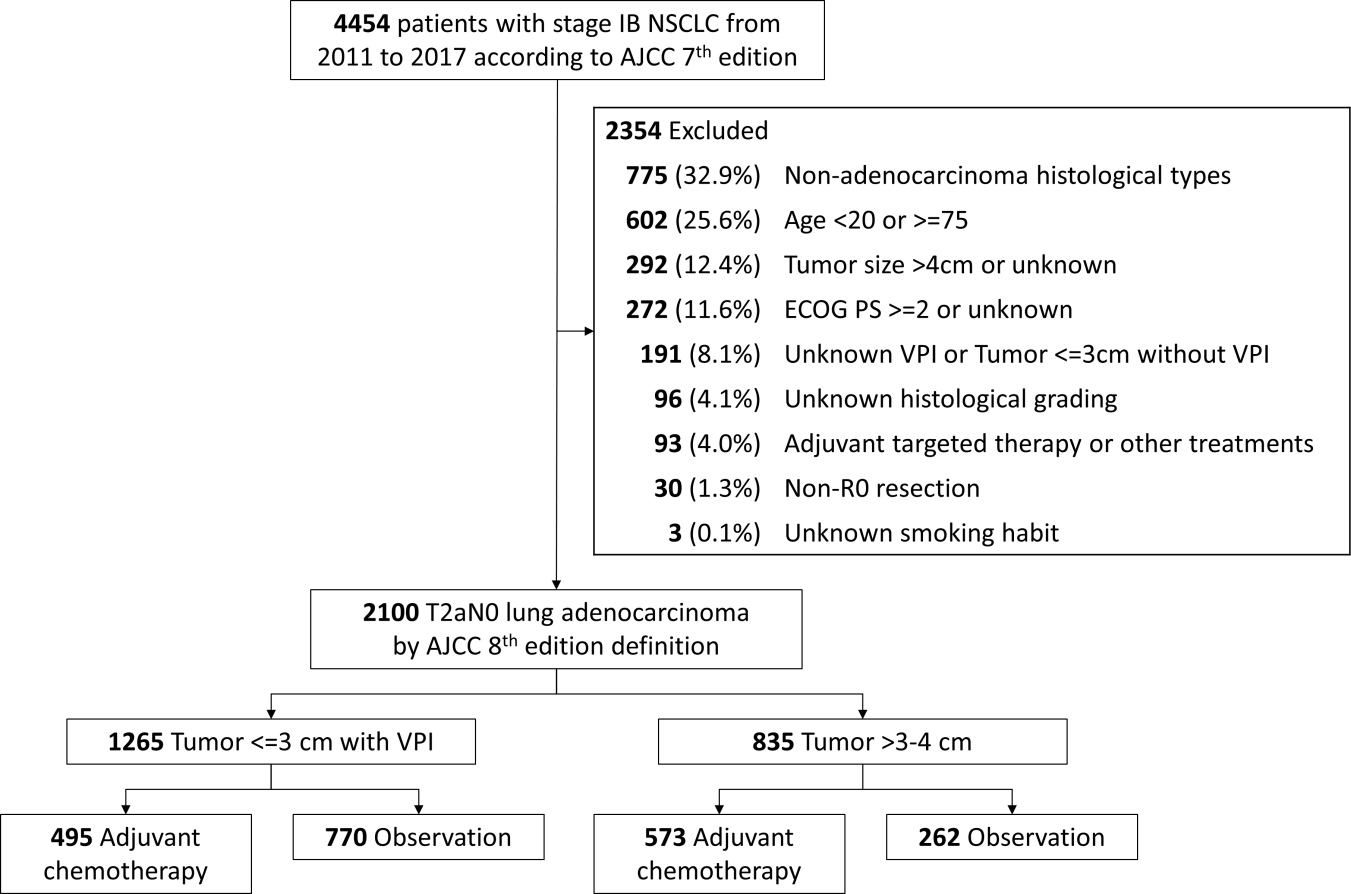
Algorithm for inclusion of study participants. Abbreviations: NSCLC, non-small-cell lung carcinoma; ECOG PS, Eastern Cooperative Oncology Group Performance Status; AJCC, American Joint Committee on Cancer; VPI, visceral pleura invasion.

We re-classified the enrolled patients according to the AJCC staging system 8^th^ edition (12). Patients who met the criteria of pathological T2aN0 stage IB were analyzed. In other words, we excluded patients with tumors larger than 4 cm. As mentioned above, patients with different histology types experienced different prognosis (18, 19), we focused on adenocarcinoma in the present study. We also excluded those who had unknown tumor size, unknown VPI, unknown histological grading, and unknown smoking status. Patients with incomplete resection of the tumor and those who received adjuvant targeted therapy or other treatments were not included. Patients younger than 20 years old, greater or equal to 75 years old, with ECOG performance status of 2 or greater were not included. The selection algorithm of participants is illustrated in **Figure 1**.

According to AJCC staging system 8^th^ edition, T2aN0 stage IB lung cancer includes tumors larger than 3 cm, but 4 cm or less in size, with involvement of main bronchus without carina, with visceral pleural invasion, or atelectasis or post obstructive pneumonitis. We categorized patients with tumors 3 cm or less into group 1. We focused on those with VPI because these populations accounted for most group 1 patients. Patients with tumors larger than 3 cm, but 4 cm or less in size were categorized into group 2.

### Statistical analyses

To compare inter-group differences for categorical and continuous variables, Pearson’s chi-square test, and t test were used respectively. Overall survival (OS) is the length of time from the date of cancer diagnosis to the date of death due to any cause, or to the date of last follow-up. Cancer-specific survival (CSS) is the length of time from the date of cancer diagnosis to the date of death from the disease. Disease-free survival (DFS) is the length of time from primary treatment for the cancer to the date of disease recurrence or death. Survival status was determined based on the national death certificate database from the Department of Statistics, Ministry of Health and Welfare, Taiwan, and the status was updated until December 31, 2020. OS and CSS of patients were estimated using the Kaplan–Meier method, whereas the inter-group differences were assessed using the stratified log-rank test. Associations between clinicopathologic variables and outcomes were assessed using Cox proportional hazards regression model. The strength of association was presented as the Hazard ratio (HR) and 95% confidence interval (CI). In this study, we used the two-tailed test, and the significant level was set at P <0.05. All analyses were performed using SAS, version 9.4 statistical software (SAS Institute Inc).

## Results

### Study population

We analyzed 2,100 patients with T2aN0 stage IB lung adenocarcinoma. There were 1,265 (60.2%) patients having tumors 3 cm or less, and 835 (39.8%) patients having tumors larger than 3 cm, but 4 cm or less in size. Amongst patients with tumors 3 cm or less, 495 (39.1%) patients received adjuvant chemotherapy, whereas patients with tumors larger than 3 cm, but 4 cm or less in size, 573 (68.6%) received adjuvant chemotherapy. The details of the patient characteristics were shown in **Supplementary Table 1**. OS and CSS were significantly different between patients with different tumor sizes (**Supplementary Figure 1A, B**). For patients in group 1 (tumors <=3 cm with VPI) and groups 2 (tumor >3 cm, but <=4 cm), the 5-year OS were 90.6% vs 84.4%, while the 5-year CSS were 93.0% vs 88.2%.

### Patient characteristics

*Group 1: tumor <=3 cm with VPI*

The characteristics of patients with tumors 3 cm or less is shown in **Table 1A**. Clinicopathological parameters were compared between the observation group and adjuvant chemotherapy group. More patients in the adjuvant chemotherapy group had high-grade histologic findings (26.7% vs. 20.9%, p=0.002), received lobectomy (86.1% vs. 76.6%, p<0.001), had larger tumors (>2 cm, but <=3 cm in size; 63.2% vs. 50.8%, p<0.001), and were treated in regional hospitals (24.8% vs. 16.5%, p<0.001). The recurrence rates between patients with and without chemotherapy were not significantly different (19.4% vs. 17.7%, p=0.418).

**Table 1A:** Patient characteristics in patients with (A) Group 1: Tumor <=3 cm with VPI, (B) Group 2: tumor >3 cm, but <=4 cm.

Table 1A. Group 1: tumor <=3 cm with VPI			
Patients, No. (%)			
Characteristic	Observation N=770	Adjuvant chemotherapy N=495	P value
Age, years			0.29
20-64	499 (64.8%)	335 (67.7%)	
65-74	271 (35.2%)	160 (32.3%)	
Sex			0.24
Male	321 (41.7%)	190 (38.4%)	
Female	449 (58.3%)	305 (61.6%)	
Histologic grade			0.02
Low	609 (79.1%)	363 (73.3%)	
High	161 (20.9%)	132 (26.7%)	
Surgery			<0.001
Sublobar resection	180 (23.4%)	69 (13.9%)	
Lobectomy	590 (76.6%)	426 (86.1%)	
N2 dissection, LN station			0.09
<3	182 (23.6%)	138 (27.9%)	
>=3	588 (76.4%)	357 (72.1%)	
Smoking habit			0.27
Ever	192 (24.9%)	110 (22.2%)	
Never	578 (75.1%)	385 (77.8%)	
ECOG			0.08
PS 0	607 (78.8%)	369 (74.5%)	
PS 1	163 (21.2%)	126 (25.5%)	
Tumor size			<0.001
<=2 cm	379 (49.2%)	182 (36.8%)	
>2-3.0 cm	391 (50.8%)	313 (63.2%)	
Hospital			<0.001
Medical left	643 (83.5%)	372 (75.2%)	
Regional hospital	127 (16.5%)	123 (24.8%)	
Tumor recurrence^#^			0.42
No	625 (82.3%)	395 (80.6%)	
Locoregional recurrence	41 (5.4%)	23 (4.7%)	
Distant recurrence	93 (12.3%)	72 (14.7%)	

PS, performance status.

^#^Patients with unknown tumor recurrence type were excluded.

*Group 2: tumor >3 cm, but <=4 cm*

The characteristics of patients with tumors larger than 3, but 4 cm or less in size are shown in **Table 1B**. Patients in the adjuvant chemotherapy group had more visceral pleural invasion (43.6% vs. 34.7%, p=0.02), more received lobectomy (95.1% vs. 90.8%, p=0.02), more with ECOG performance status of 0 (76.8% vs. 61.8%, p<0.001), and more treated in medical centers (82.4% vs. 63.0%, p<0.001). The recurrence rates between patients with and without chemotherapy were not significantly different (26.0% vs. 26.1%, p=0.517).

**Table 1B:** Group 2: tumor >3 cm, but <= 4cm

Patients, No. (%)			
Characteristic	Observation N=262	Adjuvant chemotherapy N=573	*P* value
Age, years			0.11
20-64	143 (54.6%)	346 (60.4%)	
65-74	119 (45.4%)	227 (39.6%)	
Sex			0.50
Male	106 (40.5%)	246 (42.9%)	
Female	156 (59.5%)	327 (57.1%)	
Histologic grade			0.24
Low	206 (78.6%)	429 (74.9%)	
High	56 (21.4%)	144 (25.1%)	
VPI			0.02
Absent	171 (65.3%)	323 (56.4%)	
Present	91 (34.7%)	250 (43.6%)	
Surgery			0.02
Sublobar resection	24 (9.2%)	28 (4.9%)	
Lobectomy	238 (90.8%)	545 (95.1%)	
N2 dissection, LN station			0.18
<3	59 (22.5%)	106 (18.5%)	
>=3	203 (77.5%)	467 (81.5%)	
Smoking habit			0.41
Ever	79 (30.2%)	157 (27.4%)	
Never	183 (69.8%)	416 (72.6%)	
ECOG			<0.001
PS 0	162 (61.8%)	440 (76.8%)	
PS 1	100 (38.2%)	133 (23.2%)	
Hospital			<0.001
Medical left	165 (63.0%)	472 (82.4%)	
Regional hospital	97 (37.0%)	101 (17.6%)	
Tumor recurrence^#^			0.52
No	187 (73.9%)	422 (74.0%)	
Locoregional recurrence	13 (5.1%)	40 (7.0%)	
Distant recurrence	53 (20.9%)	108 (18.9%)	

PS, performance status; VPI, visceral pleural invasion.

### Prognostic factors for survivals

*Group 1: tumor <=3 cm with VPI*

In the multivariable Cox proportional hazard model, age >65 to 74 years old, tumor size larger than 2 cm, but <=3 cm, and being treated in regional hospitals were identified as independent prognostic factors for OS (**Table 2A**). Regarding CSS, tumor size larger than 2 cm, but <=3 cm, and being treated in regional hospitals were identified as independent prognostic factors (**Table 2A**).

**Table 2A:** Univariate and multivariable analysis for (A) overall survival (OS) and cancer-specific survival (CSS) of Group 1: tumor <=3 cm with VPI; and for (B) OS and CSS of Group 2: tumor >3-4 cm.

Table 2A. Group 1: tumor <=3 cm with VPI				
Variables	Univariate analysis		Multivariable analysis^$^	
	HR (95% CI)	*P* value	aHR^*^ (95% CI)	*P* value
Overall survival:				
Age 65-74	1.54 (1.11-2.13)	0.008	1.50 (1.08-2.08)	0.016
Male	1.41 (1.03-1.94)	0.034		
High grade	1.36 (0.94-1.94)	0.097		
Sublobar resection	0.84 (0.52-1.29)	0.438		
N2 dissection, LN station <3	0.98 (0.68-1.39)	0.925		
Ever smoker	1.50 (1.05-2.11)	0.023		
ECOG PS 1	1.31 (0.91-1.85)	0.138		
Tumor size >2-3.0 cm	1.90 (1.35-2.72)	<0.001	1.83 (1.28-2.64)	0.001
Regional hospital	2.05 (1.45-2.87)	<0.001	2.13 (1.49-3.00)	<0.001
Adjuvant chemotherapy	1.10 (0.79-1.51)	0.570		
Cancer-specific survival:				
Age 65-74	1.22 (0.83-1.78)	0.290		
Male	1.24 (0.86-1.79)	0.243		
High grade	1.43 (0.94-2.13)	0.084		
Sublobar resection	0.67 (0.37-1.14)	0.166		
N2 dissection, LN station <3	0.87 (0.57-1.31)	0.526		
Ever smoker	1.28 (0.83-1.90)	0.242		
ECOG PS 1	1.12 (0.72-1.68)	0.598		
Tumor size >2-3.0 cm	2.05 (1.39-3.11)	<0.001	1.98 (1.33-3.04)	0.001
Regional hospital	2.14 (1.44-3.12)	<0.001	2.35 (1.57-3.48)	<0.001
Adjuvant chemotherapy	1.11 (0.77-1.60)	0.573		

HR, hazard ratio; aHR, adjusted hazard ratio; CI, confidence interval; PS, performance status. ^$^In multivariable analysis, only factors reaching statistical significance were listed on the table. ^*^Hazard ratio was adjusted by age, sex, histologic grade, extent of resection, smoking status, performance status, tumor size, and the types of health care institutions.

*Group 2: tumor >3 cm, but <=4 cm*

In the multivariable Cox proportional hazard model, age >65 to 74 years old, high grade histologic findings, smoking habit, and received adjuvant chemotherapy were identified as independent prognostic factors for OS. Receiving sublobar resection was identified as a prognostic factor in the univariate analysis (**Table 2B**). As regard to CSS, high grade histologic findings, receiving sublobar resection, and receiving adjuvant chemotherapy were identified as independent prognostic factors (**Table 2B**). VPI had no influence on OSS or CSS. Accordingly, we defined having either high-grade histologic findings or receiving sublobar resection as having risk factors.

**Table 2B:** Group 2: tumor >3 cm, but <=4 cm.

Variables	Univariate analysis		Multivariable analysis^$^	
	HR (95% CI)	*P* value	aHR^*^ (95% CI)	*P* value
Overall survival:				
Age 65-74	1.45 (1.07-1.98)	0.018	1.46 (1.06-2.01)	0.019
Male	1.34 (0.98-1.83)	0.062		
High grade	1.67 (1.19-2.31)	0.003	1.62 (1.14-2.28)	0.006
VPI present	1.01 (0.74-1.38)	0.947		
Sublobar resection	1.91 (1.10-3.11)	0.014		
N2 dissection, LN station <3	1.11 (0.76-1.59)	0.573		
Ever smoker	1.68 (1.22-2.30)	0.001	1.55 (1.01-2.41)	0.049
ECOG PS 1	1.33 (0.95-1.83)	0.088		
Regional hospital	1.23 (0.86-1.73)	0.242		
Adjuvant chemotherapy	0.49 (0.36-0.67)	<0.001	0.52 (0.38-0.72)	<0.001
>=1 Risk factors^*^	1.84 (1.34-2.52)	<0.001	1.73 (1.25-2.39)	<0.001
Cancer-specific survival:				
Age 65-74	1.38 (0.96-1.97)	0.079		
Male	1.32 (0.92-1.89)	0.131		
High grade	1.68 (1.13-2.45)	0.009	1.64 (1.09-2.44)	0.016
VPI present	0.93 (0.64-1.34)	0.715		
Sublobar resection	2.31 (1.26-3.89)	0.003	1.82 (0.98-3.15)	0.043
N2 dissection, LN station <3	1.26 (0.82-1.88)	0.279		
Ever smoker	1.70 (1.17-2.45)	0.004		
ECOG PS 1	1.33 (0.90-1.93)	0.142		
Regional hospital	1.24 (0.81-1.83)	0.303		
Adjuvant chemotherapy	0.50 (0.35-0.71)	<0.001	0.54 (0.37-0.78)	0.001
>=1 Risk factors^*^	1.95 (1.34-2.80)	<0.001	1.84 (1.26-2.67)	0.001

HR, hazard ratio; aHR, adjusted hazard ratio; CI, confidence interval; PS, performance status; VPI, visceral pleural invasion. ^*^ The risk factor refers to having either high-grade histologic findings or receiving sublobar resection. ^$^In multivariable analysis, only factors reaching statistical significance were listed on the table. ^*^Hazard ratio was adjusted by age, sex, histologic grade, visceral pleural invasion (VPI), extent of resection, smoking status, performance status, and the types of health care institutions.

### Association between adjuvant chemotherapy and OS or CSS

*Group 1: tumor <=3 cm with VPI*

**Figure 2:**
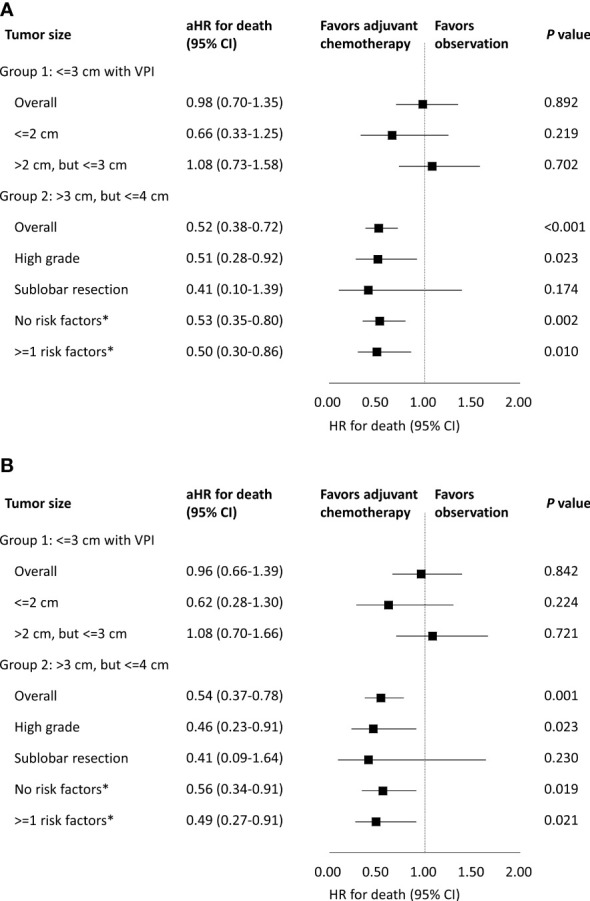
Association of **(A)** overall survival and **(B)** cancer-specific survival with adjuvant chemotherapy stratified by tumor size and risk factors. aHR, adjusted hazard ratio; CI, confidence interval; VPI, visceral pleura invasion. ^*^The risk factor in group 2 refers to having either high-grade histologic findings or receiving sublobar resection.

As shown in **Figure 2A**, amongst patients with tumors 3 cm or less with VPI, adjuvant chemotherapy was not associated with improved OS (adjusted HR [aHR], 0.98; 95% CI, 0.70-1.35; p=0.892). As mentioned previously, tumor size larger than 2 cm, but <=3 cm was identified as a prognostic factor for survival. Therefore, we sub-divided group 1 patients according to their tumor sizes (cut-off size at 2 cm), and again found no benefit of adjuvant chemotherapy on OS irrespective of tumor size. Regarding CSS, results were similar (**Figure 2B**).

**Figure 3:**
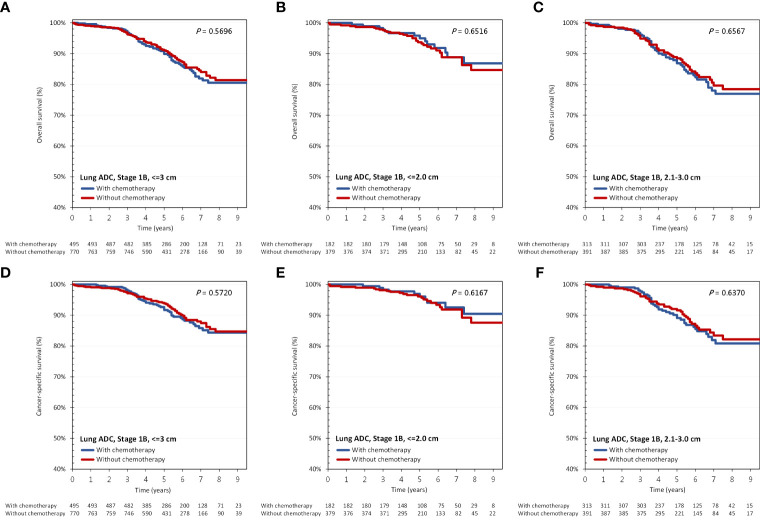
**(A–C)** overall survival and **(D–F)** cancer-specific survival according to the administration of adjuvant chemotherapy and tumor size in group 1: tumor <=3 cm with VPI. ADC, adenocarcinoma.

In group 1, OS and CSS between patients with and without adjuvant chemotherapy were similar (**Figure 3A, D**). For patients with and without adjuvant chemotherapy, their 5-year OS were 89.9% vs 91.1%, while the 5-year CSS were 91.7% vs 93.8%. In subgroup analysis according to tumor size (cut-off size at 2 cm), no survival difference was found between those with and without adjuvant chemotherapy (**Figures 3B, C, E, F**).

*Group 2: tumor >3 cm, but <=4 cm*

As shown in **Figure 2A**, in all patients with tumors larger than 3 cm, but 4 cm or less in size, adjuvant chemotherapy was associated with improved OS (aHR, 0.52; 95% CI, 0.38-0.72; p<0.001). As stated above, having either high-grade histologic findings or receiving sublobar resection were defined as risk factors in group 2. We sub-divided group 2 patients according to the presence of any risk factors, and the benefit of adjuvant chemotherapy on OS was again found even in the absence of any risk factors. Regarding CSS, results were similar (**Figure 2B**).

**Figure 4:**
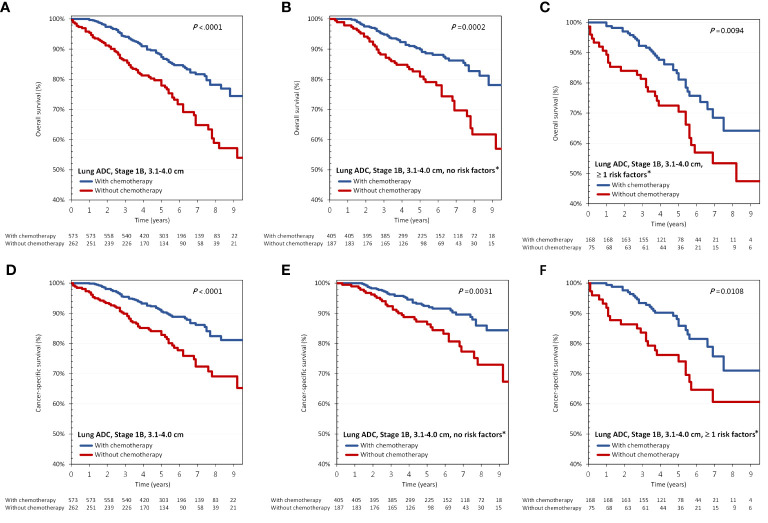
**(A–C)** overall survival and **(D–F)** cancer-specific survival according to the administration of adjuvant chemotherapy and presence of risk factors in group 2: tumor >3 cm, but <=4 cm. ADC, adenocarcinoma. ^*^The risk factor in group 2 refers to having either high-grade histologic findings or receiving sublobar resection.

In group 2, OS and CSS were significantly different between patients with and without adjuvant chemotherapy (**Figures 4A, D**). For patients with and without adjuvant chemotherapy, the 5-year OS were 87.4% vs 77.9%, while the 5-year CSS were 90.6% vs 82.8%. In subgroup analysis, the survival benefits of adjuvant chemotherapy persisted in patients with or without risk factors (**Figures 4B, C, E, F**).

### Association between adjuvant chemotherapy and DFS

In group 1, adjuvant chemotherapy did not provide DFS benefit (aHR, 0.97; 95% CI, 0.74-1.27; p=0.835). Amongst patients with tumors 2 cm or less, adjuvant chemotherapy was not associated with improved DFS (aHR, 1.00; 95% CI, 0.63-1.59; p=0.984). Amongst patients with tumors larger than 2 cm, but <=3 cm, we again found no benefit of adjuvant chemotherapy on DFS (aHR, 0.95; 95% CI, 0.68-1.33; p=0.772).

With regard to group 2, adjuvant chemotherapy was not associated with improved DFS (aHR, 0.89; 95% CI, 0.66-1.21; p=0.44). Amongst patients with risk factor, adjuvant chemotherapy was not associated with improved DFS (aHR, 0.86; 95% CI, 0.54-1.42; p=0.542). Amongst patients without risk factor, we found no benefit of adjuvant chemotherapy on DFS (aHR, 0.92; 95% CI, 0.63-1.37; p=0.675).

## Discussion

In this study, we found that the benefit of adjuvant chemotherapy was associated with tumor size amongst patients with T2aN0 stage IB lung adenocarcinoma. Adjuvant chemotherapy improved survival for those with tumors larger than 3 cm, but 4 cm or less in size. For patients with tumors 3 cm or less with VPI, adjuvant chemotherapy had no survival benefit.

Tumor size is a topic of research in predicting the benefit for resectable lung cancer patients treated with adjuvant chemotherapy. For tumors larger than 4 cm, a survival advantage has been reported in association with adjuvant chemotherapy (11, 19, 25). Therefore, we focused our study on tumors 4 cm or less without nodal involvement and found the benefit of adjuvant chemotherapy was dependent on tumor size only.

For tumors 3 cm or less with VPI, we found no survival benefit with adjuvant chemotherapy. In a previous study using the Surveillance, Epidemiology and End Results (SEER) database, adjuvant chemotherapy does not improve survival in patients with tumors 4 cm or less with VPI. However, that study did not perform exploratory analysis focusing on tumors 3 cm or less (19). Pathak et al. conducted a cohort study using data from the National Cancer Database (NCDB) of the United States to assess the association between adjuvant chemotherapy and survival in patients with node-negative early-stage NSCLC. In subgroup analysis in 2,813 patients with tumors 3 cm or less with VPI, only 297 (10.6%) had received adjuvant chemotherapy. Adjuvant chemotherapy is not associated with a survival benefit in the population (HR, 0.90; 95% CI, 0.72-1.14; p=0.38) (25). Another study using NCDB evaluated the role of adjuvant chemotherapy in patients with tumors 4 cm or less with VPI. In subgroup analysis, 6,785 patients with tumors 3 cm or less with VPI and 608 (9.0%) of them received adjuvant chemotherapy. Adjuvant chemotherapy does not provide overall survival benefit (26). Our findings are consistent with prior studies that adjuvant chemotherapy is not associated with survival benefit for tumors 3 cm or less with VPI. Compared with prior research on patients with a low adjuvant chemotherapy rate, our present study had the highest proportion (39.1%) receiving adjuvant chemotherapy for patients with tumors 3 cm or less with VPI.

For patients with tumors larger than 3 cm, but 4 cm or less in size, we found that adjuvant chemotherapy had improved their OS and CSS even in the absence of risk factors. In a cohort study based on NCDB, patients with tumors larger than 3 to 7 cm were analyzed to evaluate the role of adjuvant chemotherapy. In subgroup analysis, there were 10,587 patients with tumors >3 cm, but <=4 cm and 1,608 (15.2%) of whom had received adjuvant chemotherapy. Adjuvant chemotherapy is associated with improved OS in the population with a hazard ratio of 0.75 (95% CI, 0.70-0.86; P <0.0001) (27). In aforementioned Pathak’s study, 7,501 patients with tumors >3 cm, but <=4 cm were analyzed, and 896 (11.95%) of them had received adjuvant chemotherapy. In that population, adjuvant chemotherapy is not associated with an increase in OS (HR, 0.90; 95% CI, 0.78-1.03; p=0.21). On the other hand, adjuvant chemotherapy provides benefit only amongst patients who had received sublobar resection (HR, 0.72; 95% CI, 0.56-0.93; p=0.004) (25). In the present study, adjuvant chemotherapy was administered to 68.8% of patients with tumors >3 cm, but <=4 cm. Survival advantages in both OS and CSS were found in these patients regardless of the presence of risk factors. The difference in results across these studies may be related to differences in the studied population, chemotherapy regimen, and proportion of patients receiving adjuvant chemotherapy. Furthermore, performance status and smoking habits were not captured in the NCDB. The decision to offer adjuvant chemotherapy and survivals may be influenced by these factors. In contrast, these factors above were comprehensively recorded in the National Taiwan Cancer Registry database. Besides, CSS was unable to be evaluated in the NCDB, whereas the survival information was available in our database. With regard to the time to initiate adjuvant chemotherapy, we recommended starting adjuvant chemotherapy within 8 weeks following surgery according to prior randomized controlled trials (5, 7, 11).

The demographic characteristics of early lung cancer in Taiwan differ from that in non-Asian countries (28, 29). In our cohort, there were more female and non-smoking patients. Besides, the prevalence of *EGFR* mutation in lung cancer patients in Taiwan is higher than that in the western population (30). For patients with *EGFR*-mutant lung cancer experienced better response to EGFR-TKI or chemotherapy as compared to those with *EGFR* wild-type one if the patients suffered from disease recurrence into advanced stage (31–34), this may partly explain the discrepancy in results between our research and prior studies. Worldwide, the 5-year survival in pathologic stage IB is 73% (35), however they were 91.1% and 77.9% even in group 1 and group 2 stage IB without adjuvant chemotherapy in our study. Furthermore, according to the results from ADAURA trial, osimertinib is now standard-of-care therapy for stage IB *EGFR*-mutant lung cancer (36). This will make major improvement in survivals for stage IB *EGFR-*mutant lung cancer patients in the near future. As lung cancer is a highly heterogeneous disease, the treatment should be personalized and genetic testing could be encouraged for patients with stage IB lung adenocarcinoma. Further studies to clarify the role of driver gene mutations, immune status, and other novel treatments in adjuvant therapy following surgical resection for stage IB lung adenocarcinoma may be warranted.

There are some limitations of our study. First, it was a retrospective study. Second, the status of lymphovascular invasion, proposed as a high-risk histopathologic feature, was not recorded in the National Taiwan Cancer Registry. Third, the detailed information on adjuvant chemotherapy regimens was not collected in our cancer registry database. The regimen type, dose, and duration may influence treatment outcomes. Fourth, the treatment strategies could differ across health care institutions. Amongst patients treated in regional hospitals, the chemotherapy rates were similar between those with tumors >3 cm, but <=4 cm and those with tumors 3 cm or less (51.0% vs. 49.2%). On the other hand, in medical centers, patients with tumors >3 cm, but <=4 cm more likely to have received adjuvant chemotherapy as compared with those having tumors 3 cm or less (74.1% vs 36.7%). The inconsistency of treatment strategies across health care institutions may have introduced selection bias for adjuvant chemotherapy.

In conclusion, for patients with T2aN0 stage IB lung adenocarcinoma, the benefit of adjuvant chemotherapy depended on tumor size. Adjuvant chemotherapy within 8 weeks following surgery improved survival in those with tumors larger than 3 cm, but 4 cm or less in size. For patients with tumors 3 cm or less with VPI, adjuvant chemotherapy had no survival benefit.

## Data availability statement

The raw data supporting the conclusions of this article will be made available by the authors, without undue reservation.

## Ethics statement

This study was approved by the Research Ethics Committee of the National Taiwan University (NTU-REC No.202101HM030), with waiver of informed consent owing to the lack of personal information and use of secondary data in the study. The Strengthening the Reporting of Observational Studies in Epidemiology (STROBE) reporting guideline for observational studies was used in the revision of this article. Written informed consent for participation was not required for this study in accordance with the national legislation and the institutional requirements.

## Author contributions

Study concepts: P-HL, G-CC; Study design: C-JC, G-CC, Y-HH, J-ST; Data acquisition: C-JC, Y-HH, K-CC, K-HH; Quality control of data and algorithms: P-HL; Data analysis and interpretation: C-JC, P-HL, Z-RZ, C-HC; Statistical analysis: C-JC, W-CL; Manuscript preparation: P-HL; Manuscript editing: J-ST, T-WL; Manuscript review: G-CC, T-YY, J-YH. All authors contributed to the article and approved the submitted version.
