# Supplementary material for: Adjuvant chemotherapy compared with observation in patients with T2aN0 stage IB lung adenocarcinoma
Source: Front Oncol. 2023 Feb 28;13:1096683. doi: 10.3389/fonc.2023.1096683 (PMC10011699; doi:10.3389/fonc.2023.1096683)

Supplementary Table 1. Baseline patient characteristics

| Characteristic | Tumor <=3cm with VPI, N=1265 | Tumor >3 cm, but <=4 cm,  N=835 |
| --- | --- | --- |
| Age, years |  |  |
| 20-64 | 834 (65.9%) | 489 (58.6%) |
| 65-74 | 431 (34.1%) | 346 (41.4%) |
| Sex |  |  |
| Male | 511 (40.4%) | 352 (42.2%) |
| Female | 754 (59.6%) | 483 (57.8%) |
| Histologic grade |  |  |
| Low | 972 (76.8%) | 635 (76.0%) |
| High | 293 (23.2%) | 200 (24.0%) |
| VPI |  |  |
| Absent | 0 (0%) | 494 (59.2%) |
| Present | 1265 (100.0%) | 341 (40.8%) |
| Surgery |  |  |
| Sublobar resection | 249 (19.7%) | 52 (6.2%) |
| Lobectomy | 1016 (80.3%) | 783 (93.8%) |
| N2 dissection, LN station |  |  |
| <3 | 320 (25.3%) | 165 (19.8%) |
| >=3 | 945 (74.7%) | 670 (80.2%) |
| Smoking habit |  |  |
| Ever | 302 (23.9%) | 236 (28.3%) |
| Never | 963 (76.1%) | 599 (71.7%) |
| ECOG |  |  |
| PS 0 | 976 (77.2%) | 602 (72.1%) |
| PS 1 | 289 (22.8%) | 233 (27.9%) |
| Hospital |  |  |
| Medical center | 1015 (80.2%) | 637 (76.3%) |
| Regional hospital | 250 (19.8%) | 198 (23.7%) |
| Adjuvant chemotherapy |  |  |
| with chemotherapy | 495 (39.1%) | 573 (68.6%) |
| Observation | 770 (60.9%) | 262 (31.4%) |
| Tumor size^$^ |  |  |
| <=2 cm | 561 (44.3%) |  |
| >2-3.0 cm | 704 (55.7%) |  |
| Risk factors^*^ |  |  |
| 0 |  | 592 (70.9%) |
| >=1 |  | 243 (29.1%) |
| Tumor recurrence^#^ |  |  |
| No | 1020 (81.7%) | 609 (74.0%) |
| Locoregional recurrence | 64 (5.1%) | 53 (6.4%) |
| Distant recurrence | 165 (13.2%) | 161 (19.6%) |

Abbreviations: PS, performance status; VPI. visceral pleura invasion. ^$^The patients with tumor <=3cm with VPI were subdivided according to tumor size. ^*^ The risk factor for tumor >3-4cm refers to having either high-grade histologic findings or receiving sublobar resection. ^#^Patients with unknown tumor recurrence type were excluded.

Supplementary Figure 1 (A) overall survival and (B) cancer-specific survival according to tumor size.


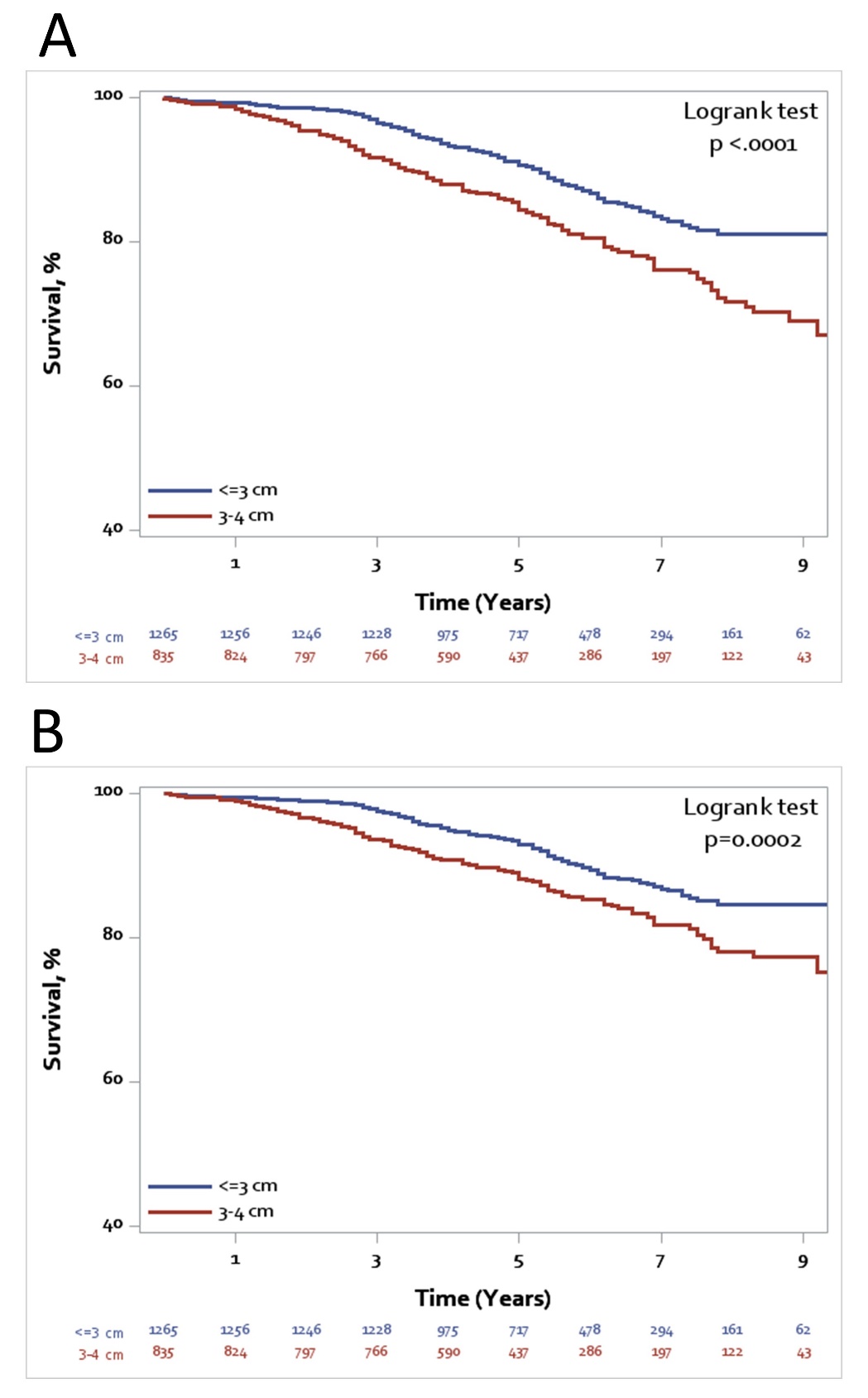

Supplement: Supplementary file 1 [file DataSheet_1.docx]
